# Mediating effects of self-concept clarity and self-objectification on the relationship between celebrity worship and the process of considering cosmetic surgery among Chinese undergraduates

**DOI:** 10.1186/s40359-022-00975-6

**Published:** 2022-11-10

**Authors:** Jia Cui, Yong Fang

**Affiliations:** 1 Department of Student Affairs, Wuhan University of Science and Technology, Wuhan, China; 2 School of Nursing, Hubei University of Chinese Medicine, Wuhan, China

**Keywords:** Celebrity worship, Cosmetic surgery consideration, Self-concept clarity, Self-objectification

## Abstract

**Background:**

Several studies have examined the relationship between celebrity worship and cosmetic surgery; however, few have discussed the mediating role of self-concept. To fill this research gap, the present study aims to examine the mediating roles of self-concept clarity and self-objectification in the association between celebrity worship and cosmetic surgery.

**Methods:**

A sample of 1,089 Chinese undergraduates (*M*_age_ = 20.32; *SD*_age_ = 2.60) completed measures of celebrity worship, actively considering cosmetic surgery, self-concept clarity, and self-objectification. Mediating effect analysis was used to test the hypothesis.

**Results:**

The results showed that celebrity worship, cosmetic surgery consideration, and self-objectification were positively correlated, whereas self-concept clarity was negatively correlated with all three variables. Mediation analysis revealed that celebrity worship predicted consideration of cosmetic surgery not only directly but also through three indirect paths through the mediating role of (1) self-concept clarity, (2) self-objectification, and (3) the chain mediating role of self-concept clarity and self-objectification.

**Conclusions:**

These findings broaden our understanding of the psychological processes that underlie the association between celebrity worship and considering cosmetic surgery and afford practical guidance on reducing the risks associated with cosmetic surgery.

## Background

“Her robe is made of cloud, her face of flowers made; Caressed by vernal breeze, freshened by morning dew; Charming as Fairy Queen in her Mountain of Jade; Or Goddess of the Moon in her palace sky-blue.” This is a poem about beauty written more than a thousand years ago by the Chinese famous poet Li Bai. Through the ages, Chinese people have wanted to express their yearning and praise for beauty. With the development of economic growth and medical technology, more and more Chinese people are choosing to optimize their appearance through cosmetic surgery. In a survey of 637 Chinese undergraduates on their attitudes toward cosmetic surgery, 32.3% said that they were willing to have cosmetic surgery, and they believed that cosmetic surgery could boost their confidence and have a positive impact on job hunting and dating [1]. According to China cosmetic surgery industry development prospect and investment risk forecast analysis report, the scale of China's cosmetic surgery market increased from 52 billion yuan to 151.8 billion yuan from 2014 to 2019, becoming the second largest cosmetic surgery market in the world [2]. Although more and more people are opting for cosmetic surgery, some potential risks are easily overlooked, such as chronic numbness of facial expression, chronic pain, and infections [3–5]. Additionally, cosmetic surgery is associated with some negative psychological problems, such as depression, body dysmorphic disorders, and social isolation [6, 7]. Given the increasing number of undergraduates choosing cosmetic surgery as a strategy for changing their appearance and the potential risks of cosmetic surgery, it is now necessary to develop a better understanding of the psychological mechanisms involved in actively pursuing cosmetic surgery.

### Celebrity worship and cosmetic surgery

Sociocultural factors play an important role in the development of attitudes toward cosmetic surgery. Sociocultural models were originally used to explain the occurrence of negative body image and eating disorders [8], most notably the three-factor model proposed by Thompson [9]. The three-factor model suggests that media, peers, and parents are important sources of body image and eating disorders. Researchers found that higher media exposure was associated with a higher willingness to undergo cosmetic surgery [10]. Attachment to media figures is considered an important aspect of media influence, which can shape the cognition of adolescents and young adults [11]. The media offer the main platform for celebrities to show themselves. A study revealed that 54.4% of 1729 Chinese undergraduates are celebrity worshipers and that entertainment stars account for 64.1% of Chinese undergraduates’ favorite celebrities [12].

According to the absorption-addiction model by McCutcheon et al., celebrity worship is an excessive admiration towards a famous person, and celebrity worship can be considered as a continuum ranging from a healthy enthusiasm to compulsive behaviors and pathological feelings towards a favorite celebrity [13]. Empirical studies have shown that celebrity worship is associated with poor self-image and eating disorders [14–16]. Swami et al.’s study on 401 British female undergraduates found that celebrity worship was positively correlated with the degree of subsequent acceptance of cosmetic surgery [10]. Maltby and Day found that the strong worship of physically superior celebrities predicted the incidence of cosmetic surgery [17].

### The mediating roles of self-concept clarity and self-objectification

Researchers believe that celebrity worship is an important part of self-development and will necessarily affect self-development [11, 13]. As a core part of individual cognitive system, self-concept is an individual’s perception judgment of self-ability and value based on existing experience [18]. Self-concept clarity refers to the degree to which individuals are clear and confident about the content of self-concept, the consistency between the content of their own self-concept, and the stability over time dimension, which is both a kind of trait and a state [19]. The absorption addiction model states that high levels of celebrity worship lead to impaired self-structure and greater identification with celebrities, in an attempt to establish a self-image [13]. Reeves et al. found that celebrity worship was associated with lower self-concept clarity [20]. People with low self-concept clarity are more likely to internalize external standards as a means of defining themselves [21]. Several related studies have shown that low self-concept clarity is associated with greater internalization of idealizing thinness as a virtue and attendant greater body dissatisfaction [22–24]. Those with lower self-concept clarity made more intense comparisons when viewing idealized images, leading to greater body dissatisfaction after exposure [25]. Therefore, it is inferred that the higher the degree of celebrity worship, which may lead to the loss of self-concept clarity, the more likely the consideration of cosmetic surgery.

Self-objectification means that individuals internalize a third-party perspective and regard their own body as the object of appearance-based evaluation, which is not only a persistent and stable trait but also a state that can be triggered by the situation pertaining [26]. Affected by social environment and media, people will focus on their appearance and regard it as an object that can be evaluated, thereby resulting in self-objectification [26, 27]. Celebrities are a group of people who are active in the media. Maltby et al. showed a significant correlation between strong celebrity worship and concern for body shape [28]. We may thus infer that celebrity worship among undergraduates is likely to lead to self-objectification. Self-objectification is linked to several negative outcomes, such as mental health problems, eating disorders, and impaired cognition [29]. According to the objectified model of cosmetic surgery, people’s desire to improve their appearance often stems from an objectified view of themselves [30]. Studies have shown that self-objectification is an important predictor of individuals’ propensity for cosmetic surgery [31, 32].

Self-concept clarity belongs to the structural level of self-concept that refers to the organization of self-related components [19], whereas self-objectification belongs to the content level. As has been indicated, the structure of a system would affect its content [33]. Kong et al. verified that self-concept clarity positively predicts self-esteem [34], which also belongs to the content level [35]. The lower the level of clarity, the more likely the self-concept will change [36]. Thus, self-concept clarity may affect self-objectification.

## The present study

In summary, the available researches have shown that celebrity worship is markedly associated with actively considering cosmetic surgery; however, there are unresolved issues and gaps in knowledge vis-à-vis mediating pathways. This study aims to explore the mechanism by which celebrity worship leads to actively considering cosmetic surgery.

**Fig. 1:**
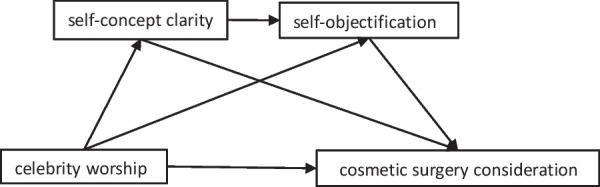
Hypothesized conceptual model

Based on the discussions above, self-concept clarity and self-objectification may help explain the influence of celebrity worship on cosmetic surgery consideration. What is more, we suspect that self-concept clarity and self-objectification may work together through chain mediation. The present study constructed a multiple mediation model (see Fig. 1) to examine the mediating roles of self-concept clarity and self-objectification between celebrity worship and cosmetic surgery among Chinese undergraduates. We proposed the following hypotheses:

### **Hypothesis 1**

Celebrity worship positively predicts actively considering cosmetic surgery among Chinese undergraduates.

### **Hypothesis 2**

Self-concept clarity plays a mediating role in the effect of celebrity worship on actively considering cosmetic surgery.

### **Hypothesis 3**

Self-objectification plays a mediating role in the effect of celebrity worship on actively considering cosmetic surgery.

### **Hypothesis 4**

Self-concept clarity and self-objectification play a chain mediating role in the effect of celebrity worship on actively considering cosmetic surgery.

## Methods

### Participants

The present study was conducted among Chinese undergraduates with the approval of the Ethics Committee of Wuhan University of Science and Technology. The questionnaire was delivered online through the Wenjuanxing platform, and the questionnaire links were published at two full-time universities in Wuhan, China. Further, 1,262 participants completed the questionnaire, of which 173 were excluded as they failed any of the validity checks included in this study (i.e., questions asking participants to select a specific response option). The final sample comprised 1,089 participants. There were 634 males and 455 females. Regarding age, participants ranged from 18 to 24 years, and the mean age was 20.32 years (SD = 2.60).

## Measures

### Celebrity worship

Celebrity worship was assessed using the Chinese Revised Version of Celebrity Attitude Scale revised by Peng et al. [37]. The scale consists of 27 items (e.g., “I pay special attention to the details of my favorite celebrity’s life”), including five dimensions of recreational social interaction, emotional projection, complete identity, relational fantasy, and pathological edge. The scale is rated on a 5-point Likert scale (1 = not at all to 5 = absolutely). The higher the score, the higher the level of celebrity worship. In the present study, the internal consistency coefficient of the scale was 0.96. The model-data fit indexes for the 27-item five-factors EFA model showed an acceptable fit: RMSEA = 0.08, NFI = 0.87, CFI = 0.88, TLI = 0.87.

### Cosmetic surgery consideration

Following Calogero et al. [30], the five-item Acceptance of Cosmetic Surgery Scale was the dependent measure in this study. It assessed the extent to which people were willing to consider cosmetic surgery in the future (e.g., “I sometimes want to have cosmetic surgery”) and the factors that influenced this decision (e.g., cost, pain, and side effects). Zero to 6 points are scored from “strongly disagree” to “strongly agree.” The higher the score, the more likely the individual is to have cosmetic surgery. Jackson and Chen conducted a confirmatory factor analysis on Chinese undergraduates; the results showed that the scale was suitable for Chinese undergraduates, and the reliability and validity of the Chinese version of the scale were good [38]. In the present study, the internal consistency reliability of the scale was 0.85.

### Self-concept clarity

Self-concept clarity was measured by the Self-Concept Clarity Scale developed by Campbell et al. [19]. The scale contains 12 items that measure the clarity and consistency of an individual’s self-perception, such as “It is difficult for me to make up my mind to do something because I do not know what I really want”. The scale adopts a 5-point scoring system, ranging from 1 = strongly disagree to 5 = strongly agree. The higher the sum of the measurement results, the higher the definition of individual self-concept. In the present study, the internal consistency reliability of the scale was 0.81.

### Self-objectification

Self-objectification was measured via the Body Surveillance Subscale of the Objectified Body Consciousness Scale developed by McKinley and Hyde [39]. The scale included eight items to measure how concerned individuals were about their body appearance (e.g., “I think about how I look several times a day”). A 7-point scale was used, ranging from “1 = completely disagree” to “7 = completely agree.” Higher scores indicate more frequent physical monitoring, more attention to the body, and higher levels of self-objectification. In the present study, the internal consistency coefficient of the scale was 0.80.

### Statistical analysis

Descriptive statistical analysis, ANOVA analyses and correlation analysis were conducted on the collected data using SPSS Statistics version 21.0. Then, we used the PROCESS macro in SPSS, developed by Hayes [40], for multiple mediation analyses. The present study utilized the PROCESS macro Model 6 to test the roles of self-concept clarity and self-objectification in mediating the association between celebrity worship and actively considering cosmetic surgery. Through repeated random sampling, 5,000 bootstrap samples were selected, and the bootstrap 95% confidence interval of the mediation effect was calculated. Considering that gender was associated with celebrity worship and cosmetic surgery [41, 42], it was included as a control variables in the statistical analyses.

## Results

### Correlational analyses

Correlation analysis showed that celebrity worship, self-concept clarity, self-objectification, and actively considering cosmetic surgery were correlated with each other (Table 1 shows the mean value, standard deviation, and correlation coefficients of each variable). All research variables were significantly correlated in the predicted directions, which is suitable for further analysis.

**Table 1:** Correlations between variables (*N* = 1,089)

	*M*	*SD*	1	2	3	4
1. Celebrity worship	50.31	20.74	1			
2. Self-concept clarity	34.27	7.51	− 0.14**	1		
3. Self-objectification	30.01	8.26	0.15**	− 0.13**	1	
4. Cosmetic surgery consideration	12.14	6.80	0.14**	− 0.14**	0.34**	1

*P < 0.05, **P < 0.01, ***P < 0.001, which also apply to the following tables

### Mediation effect testing

As shown in Table 2, after standardizing all the variables, celebrity worship was a significant predictor of actively considering cosmetic surgery after controlling for gender (*β* = 0.36, *p* < 0.001). Celebrity worship significantly negatively predicted self-concept clarity (*β* =  − 0.14, *p* < 0.001). When both celebrity worship and self-concept clarity predicted self-objectification, both celebrity worship and self-concept clarity showed significant predictive effects (*β* = 0.11, *p* < 0.01; *β* =  − 0.09, *P* < 0.01). Celebrity worship, self-concept clarity, and self-objectification also predicted cosmetic surgery consideration significantly (*β* = 0.08, *p* < 0.01; *β* =  − 0.09, *p* < 0.01; *β* = 0.29, *p* < 0.001).

**Table 2:** Results of the multiple mediation analysis

Outcome variable	Independent variable	*R*^2^	*F*	*β*	*t*
Cosmetic surgery consideration	Gender	0.05	29.02	0.36	5.88***
	Celebrity worship			0.13	4.10***
Self-concept clarity	Gender	0.02	11.54	0.06	1.02
	Celebrity worship			− 0.14	− 4.76***
Self-objectification	Gender	0.06	22.58	0.34	5.73***
	Celebrity worship			0.11	3.63**
	Self-concept clarity			− 0.12	− 3.57**
Cosmetic surgery consideration	Gender	0.14	47.25	0.27	4.56***
	Celebrity worship			0.08	− 3.11**
	Self-concept clarity			− 0.09	− 3.11**
	Self-objectification			0.29	9.73***

The percentile bootstrap method with bias correction was further used to test the mediation effect. As shown in Table 3, the 95% confidence interval of the direct effect does not include 0, indicating that the direct effect is significant. The 95% confidence interval of the intermediary path does not include 0, indicating that the mediation effects are significant. It suggests that self-concept clarity and self-objectification play a partial mediating role between celebrity worship and actively considering cosmetic surgery.

**Table 3:** Bootstrap analysis of multiple mediation effects

Model pathways	Effect size	LLCI	ULCI	Effect (%)
Celebrity worship → cosmetic surgery consideration (total effect)	0.126	0.07	0.19	100
Celebrity worship → cosmetic surgery consideration (direct effect)	0.076	0.02	0.14	60.32
Celebrity worship → self-concept clarity → cosmetic surgery consideration	0.012	0.004	0.024	9.52
Celebrity worship → self-objectification → cosmetic surgery consideration	0.033	0.016	0.053	26.19
Celebrity worship → self-concept clarity → self-objectification → cosmetic surgery consideration	0.005	0.002	0.009	3.97

## Discussion

The present study examined the relationship between celebrity worship and actively considering cosmetic surgery with the mediating effects of self-concept clarity and self-objectification among Chinese undergraduates. The results showed that celebrity worship was significantly positively correlated with actively considering cosmetic surgery, which verified H1 and was consistent with previous findings [10, 17]. Swami et al. argued that argued that celebrities provide information about beauty standards that are internalised and aspired to [10]. According to the absorption-addiction model, worshipers will invest a large amount of cognitive resources in celebrities, and they will feel a special connection with celebrities and adopt celebrities as role models to establish themselves [13]. If undergraduates immerse themselves in visual and audio works for a long time, they are likely to internalize the appearance conditions of celebrities as their own standards of appearance and desire to be like their favorite celebrities [8]. Thus, they are more willing to change their appearance to meet these standards, which leads to greater consideration of having cosmetic surgery.

The results revealed that celebrity worship not only predicted actively considering cosmetic surgery directly but also had an indirect effect on three specific paths. First, celebrity worship predicted actively considering cosmetic surgery through the mediating effect of self-concept clarity, thus supporting H2. In other words, celebrity worship would lead to poorer self-concept clarity, and the poorer the self-concept clarity, the higher the cosmetic surgery consideration. High levels of celebrity worship affect self-concept [28]. The life status, behavior style, values, and other factors of celebrity will impact individuals’ self-concept and affect the stability of the self-concept. Self-concept clarity is closely related to psychosocial adaptation. Related studies have found that generally, individuals with low self-concept clarity are more sensitive and vulnerable than others [43] and more likely to make social comparisons [24]. Undergraduates whose self-concept is unstable and unclear are more likely to compare their appearance to those of celebrities and be dissatisfied with their appearance, thus considering cosmetic surgery more seriously.

Second, self-objectification mediated the relationship between celebrity worship and actively considering cosmetic surgery, verifying H3. According to the theory of self-objectification, people will regard their own bodies as evaluable objects under the influence of the environment, media, and other factors. The celebrities in the media make people pay more attention to their bodies [28]. Self-objectification is positively correlated with appearance anxiety [44, 45]. People with high levels of self-objectification were more likely to compare their appearance to others [46, 47]. Upward comparison in the media is an important predictor of actively considering cosmetic surgery [48]. In short, celebrities are active in the media with their outstanding appearance, which makes fans pay more attention to their appearance, which, in turn, leads to greater active consideration of cosmetic surgery.

Finally, the results of this study also support the chain-mediated hypothesis that celebrity worship can indirectly predict individuals’ cosmetic surgery consideration through self-concept clarity and self-objectification. The result indicated that fanatical celebrity worshipers had low levels of self-concept clarity, which subsequently increased their levels of self-objectification and finally increased their consideration of cosmetic surgery. According to identity theory, the development of self-identity is an important process for young adults [49]. Influenced by celebrity worship, undergraduates’ self-concept clarity becomes unstable [13]. As is known, self-concept clarity is an important factor for individuals’ subjective well-being and psychosocial adaptation, especially for young adults whose identity begins to consolidate [49, 50]. When their sense of self is uncertain, they tend to focus too much on their body image, leading to body dissatisfaction and actively considering cosmetic surgery. This suggests that mediating models with multiple self-variables more fully reveal the relationship between celebrity worship and considering cosmetic surgery.

We believe that the present study has broadened our knowledge of the mechanisms linking celebrity worship to actively considering cosmetic surgery among Chinese undergraduates and verified the important role of self-concept. The findings add to a growing body of research into celebrity worship and cosmetic surgery and the role of the self in both. The results suggest that undergraduates’ excessive celebrity worship may be harmful to them, with the possibility of perhaps pathological cosmetic surgery. Undergraduates should be guided to worship celebrities rationally and moderately. Our findings indicate that concept clarity and self-objectification are influence factors for cosmetic surgery among Chinese undergraduates. Consequently, to minimize the negative impact of cosmetic surgery, teachers should help undergraduates establish a clear self-concept and avoid adopting their own body as an evaluation object.

The present study has some shortcomings and needs to be further improved. First, although our participants were enrolled from two separate universities in central China, they may not be fully representative of all undergraduates in China. Future studies can expand the sample to explore the effects of demographic variables such as region and ethnic factors on celebrity worship and cosmetic surgery. Second, as the results are based on cross-sectional data, causal relationships cannot be inferred and the findings should be treated with caution. Future studies could consider using longitudinal designs to prove causality. Third, the data were collected through self-reporting questionnaires, which may be subject to various biases. Collecting data from multiple sources can minimize biasing effects.

Results indicated that self-concept clarity and self-objectification not only mediated the relationships between celebrity worship and actively considering cosmetic surgery separately, but their chained mediating path was also significant. These findings verified our hypotheses and extended the previous research results by explaining how celebrity worship is related to actively considering cosmetic surgery.

## Data Availability

The datasets used and/or analysed during the current study will be available from the corresponding author on reasonable request.

## Abbreviations

EFA: Exploratory factor analysis
RMSEA: Root mean square error of approximation
CFl: Comparative fit index
NFI: Normal of fit index
CFI: Comparative fit index
TLI: Tucker–Lewis index
ANOVA: Analysis of variance
